# Supercritical Fluid Chromatography for Expedited Sample Clean‐Up in Isotope Ratio Mass Spectrometry Including Cholesterol as New Endogenous Reference Compound

**DOI:** 10.1002/dta.70150

**Published:** 2026-09-20

**Authors:** Thomas Piper, Jana Drewes, Mario Thevis

**Affiliations:** ^1^ German Sport University Cologne Center for Preventive Doping Research – Institute of Biochemistry Köln Germany; ^2^ University of Münster Institute of Inorganic and Analytical Chemistry Münster Germany; ^3^ European Monitoring Center for Emerging Doping Agents (EuMoCEDA) Cologne/Bonn North Rhine‐Westphalia Germany

**Keywords:** cholesterol, doping controls, fraction collection, isotope ratio mass spectrometry, supercritical fluid chromatography

## Abstract

Doping control samples showing elevated urinary concentrations and concentration ratios of testosterone or testosterone metabolites are forwarded to isotope ratio mass spectrometry‐based determinations in order to differentiate between naturally elevated concentrations and doping offenses. Routine sample preparation protocols commonly encompass liquid–liquid and solid‐phase extraction steps and enzymatic deconjugation of steroid glucuronides. In order to separate all steroids of interest from matrix components and to obtain sufficiently clean urinary extracts, high performance liquid chromatography (HPLC) with fraction collection is a frequently employed method of choice. This preparation step is time consuming as each HPLC run requires approx. 45 min, and the evaporation of collected fractions containing water and acetonitrile necessitates up to 60 min. Supercritical fluid chromatography employs supercritical carbon dioxide as eluent combined with other organic solvents as modifiers. The unique physiochemical properties of supercritical carbon dioxide enable the acceleration of the chromatographic separation of different compounds, and collected fractions only contain the modifier and additional methanol as make‐up solvent, which allows for rapid evaporation within 10 min. Therefore, a method was developed and validated in‐line with current World Anti‐Doping Agency‐stipulated regulations encompassing testosterone, epitestosterone (E), dehydroepiandrosterone, androsterone, etiocholanolone, 5α‐ and 5β‐androstanediol as target analytes and cholesterol, pregnanediol, 16‐androstenol, and 11‐oxo‐etiocholanolone as endogenous reference compounds. Reference population data encompassed 40 female and 40 male routine doping control samples. Cholesterol as a new endogenous reference compound was carefully investigated. As proof‐of‐concept, samples derived from a testosterone‐gel administration trial were investigated, demonstrating an impact of the drug intervention on E.

## Graphical abstract

Supercritical fluid chromatography was employed in the sample preparation for IRMS determinations in sports drug testing for the first time and the developed method was successfully validated. Cholesterol (CHOL) was introduced as a novel endogenous reference compound and population‐based Δ‐values were established as shown here. Abbreviations: 5aDIOL, 5α‐androstanediol; 5bDIOL, 5β‐androstanediol; A, androsterone; DHEA, dehydroepiandrosterone; E, epitestosterone; ETIO, etiocholanolone; T, testosterone.

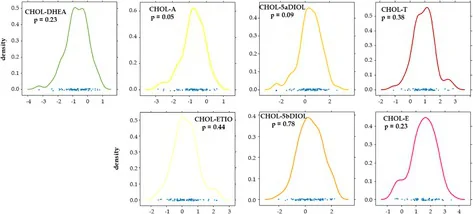

## 1 Introduction

The detection of testosterone (T) doping remains challenging for doping control laboratories as T represents an endogenous compound, i.e., it is naturally present in all urine samples. In order to detect the illicit administration of T and T‐prohormones, the quantification of T and T‐metabolites is necessary in every urine sample [1]. Elevated urinary concentrations are identified via diagnostic ratios like T over epitestosterone (E) and monitored in a longitudinal manner in the Steroidal Module of the Athlete Biological Passport [2]. If ratios exceed the individual thresholds, samples are forwarded to isotope ratio mass spectrometry (IRMS) in order to distinguish between naturally elevated or by confounding factors affected concentrations and doping offenses. This differentiation is based on the determination of carbon isotope ratios (CIR) in target analytes (target compounds, TCs) like T or T‐metabolites and the comparison of these CIR with those of endogenous reference compounds (ERCs). If the found differences between ERCs and TCs—so called Δ‐values—fall beyond established thresholds, the exogenous origin of T or T‐metabolites has to be considered [3].

In order to enable precise CIR determinations in IRMS, all analytes are thermally converted to CO_2_ prior to measuring. This procedure requires pure chromatographic peaks of each analyte not affected by any co‐eluting substance to ensure valid results [4, 5]. Based on this prerequisite, sophisticated sample preparation protocols are necessary, encompassing—besides solid‐phase and liquid–liquid extractions—high performance liquid chromatography (HPLC) with fraction collection as the core part of sample clean‐up [6, 7, 8, 9, 10]. The HPLC‐based isolation of steroids from the biological urinary matrix takes between 45 and 90 min depending on the chosen protocol followed by evaporation of each collected fraction which again depending on the percentage of water in the fraction, lasts for up to 60 min. Employing supercritical fluid chromatography (SFC) instead of conventional HPLC should considerably speed up this part of sample preparation.

Instead of using water and an organic solvent as eluents, the chromatographic separation in SFC is based on supercritical CO_2_ in combination with an organic solvent as modifier [11, 12]. Although SFC has already been used in combination with mass spectrometry for decades, fraction collection remained complicated due to the physical properties of CO_2_, expanding very fast after eluting from the pressurized SFC system. In order to overcome these limitations, a fraction collection system was developed especially for SFC‐based HPLC systems and employed in this study for the first time in the context of doping controls [13].

In line with current regulations of the World Anti‐Doping Agency (WADA), a novel method was developed and validated fulfilling all necessary requirements for sports drug testing [3]. Besides T and E, androsterone (A), etiocholanolone (ETIO), 5α‐androstane‐3α,17β‐diol (5aDIOL), 5β‐androstane‐3α,17β‐diol (5bDIOL) and dehydroepiandrosterone (DHEA) were employed as TCs whereas pregnanediol (PD), 16‐androstenol (16EN), 11‐oxo‐etiocholanolone (11‐oxo‐ETIO) and cholesterol (CHOL) were chosen as ERCs. Besides being the metabolic precursor of all endogenous steroids, urinary CHOL has until now only been used sparsely as ERC but was recently investigated more detailed in human serum samples as a potential alternative ERC [14, 15, 16, 17]. The source of urinary CHOL was under discussion, i.e., if the found CHOL was derived from cells from the urinary tract or represents CHOL from the circulation excreted in parallel with other TCs and ERCs, and remained questionable excluding this steroid as ERC in doping controls so far [18]. As the results in serum were promising and allowed for calculating sensitive and reliable population‐derived thresholds for Δ‐values, the suitability of urinary CHOL as ERC was investigated here by comparison of the CIR of CHOL extracted from urine to CHOL extracted from dried blood spots (DBS) that were collected from the same individuals included in the reference population at the same time. This experiment may enable to prove that excreted CHOL shows comparable CIR to circulating CHOL, which would allow for using CHOL as a novel ERC.

## 2 Experimental

### 2.1 Chemicals and Steroids

Steroid reference material 3β‐hydroxy‐5α‐androstane (RSTD), 5aDIOL, and 5bDIOL were purchased from Steraloids (Newport, Rhode Island, USA). Steroid reference material CHOL, PD, 16EN, 11‐oxo‐ETIO, A, ETIO, DHEA, T, E, and methyltestosterone (MeT) were from Sigma Aldrich (Steinheim, Germany). Methanol (MeOH), *tert*‐butyl methyl ether (TBME), acetonitrile (ACN), ethanethiol, and ammonium iodide (NH_4_I) were purchased from Merck (Darmstadt, Germany). *N*‐methyl‐*N*‐trimethylsilyltrifluoroacetamide (MSTFA) was purchased from Chemische Fabrik Karl Bucher (Waldstetten, Germany). All solvents and reagents were of analytical grade. The β‐glucuronidase from *Escherichia coli* employed for enzymatic hydrolysis was from Roche Diagnostics GmbH (Mannheim, Germany). Solid‐phase extraction (SPE) cartridges Chromabond C18 (500 mg) were obtained from Macherey & Nagel (Düren, Germany). Helium (purity 5.0), oxygen (purity 5.0) and the CO_2_ used for the SFC (purity 2.5, riser pipe bottle) were from Linde (Pullach, Germany). The CO_2_ tank gas (purity 4.5, Linde) used in IRMS was calibrated against the secondary reference material USADA 33‐1 provided by Cornell University (Ithaca, New York, USA) [19]. The calibration was performed periodically following the recommendations of the applicable TD IRMS, i.e., after cleaning the ion source or changing the analytical GC column or the combustion tube [3].

The acetic anhydride used for acetylation was from Sigma Aldrich and stored over a molecular sieve (4A beads, mesh 4‐8, Sgma Aldrich) to enhance and maintain good acetylation yields. The isotopic composition necessary for accurate corrections was established once using secondary reference material USADA 34 (Cornell University) [19].

### 2.2 Sample Preparation

The sample preparation was adapted from already published protocols [6, 20]. In brief, depending on the urinary concentration of endogenous steroids, up to 20 mL of urine was applied in portions to a pre‐conditioned SPE cartridge and washed with 2 mL of water. Then, the SPE was eluted with 3 mL of MeOH and evaporated to dryness under a stream of air at 50°C. The dried residue was reconstituted in 2 mL of phosphate buffer (pH 7) and incubated in a heating block for 60 min after adding 100 μL of β‐glucuronidase. After cooling to room temperature, 1 mL of carbonate buffer (pH 10) was added and formerly unconjugated and glucuronidated steroids were extracted adding 5 mL of TBME. The organic layer was transferred to a new test tube, evaporated to dryness, and then the residue was transferred to an auto‐sampler vial using 200 μL of ACN. The sample was dried down in a desiccator and finally reconstituted in 40 μL of ACN for the injection in the SFC system.

### 2.3 SFC‐Based Clean up

In order to achieve the purity of all analytes necessary for valid IRMS determinations, all samples were purified using a Shimadzu Nexera UC Prep Multi‐Fraction System (Shimadzu Deutschland GmbH, Duisburg, Germany). The system essentially consisted of a supercritical fluid pump, a back‐pressure regulator, an autosampler, two solvent delivery pumps, a column oven, and a photodiode array detector. The core part of the system to enable its use in sample preparation was the fraction collector FRC‐40 equipped with a LotusStream Separator [13].

Numerous columns, modifiers, and gradients were tested within this research project, and the final approach relied on an Agilent InfinityLab Poroshell 120 HILIC (4.6 mm, 250 mm, 4 μm) column from Agilent (Waldbronn, Germany) protected by an EC 4/3 Nucleodur HILIC 3 μm pre‐column (Macherey&Nagel, Düren, Germany). The modifier was ACN, and MeOH was used as make‐up solvent for fraction collection at a constant flow of 0.7 mL/min. The chromatographic flow was at 3.5 mL/min starting with 98% CO_2_, changing to 75% CO_2_ in 16 min, held for 6 min at this level, followed by 2 min of 60% CO_2_ before re‐equilibration at 98% CO_2_ for 2 min, resulting in a run time of 26 min for each injection including the re‐equilibration. Injection volume was 40 μL using ACN as solvent. Fractions were collected as follows and a standard chromatogram is depicted in Figure 1: F1 from 5.7 to 6.5 min (16EN), F2 from 7.1 to 8.0 min (CHOL), F3 from 11.00 to 11.70 min (DHEA), F4 from 12.40 to 13.20 min (A), F5 from 14.40 to 16.00 min (T, ETIO, and 5aDIOL), F6 from 16.90 to 17.70 min (E), F7 from 18.80 to 19.55 min (5bDIOL), and F8 from 19.56 to 20.80 min (PD and 11‐oxo‐ETIO). All fractions were collected in test‐tubes and evaporated to dryness in a heating block at 50°C using air in less than 10 min.

**FIGURE 1:**
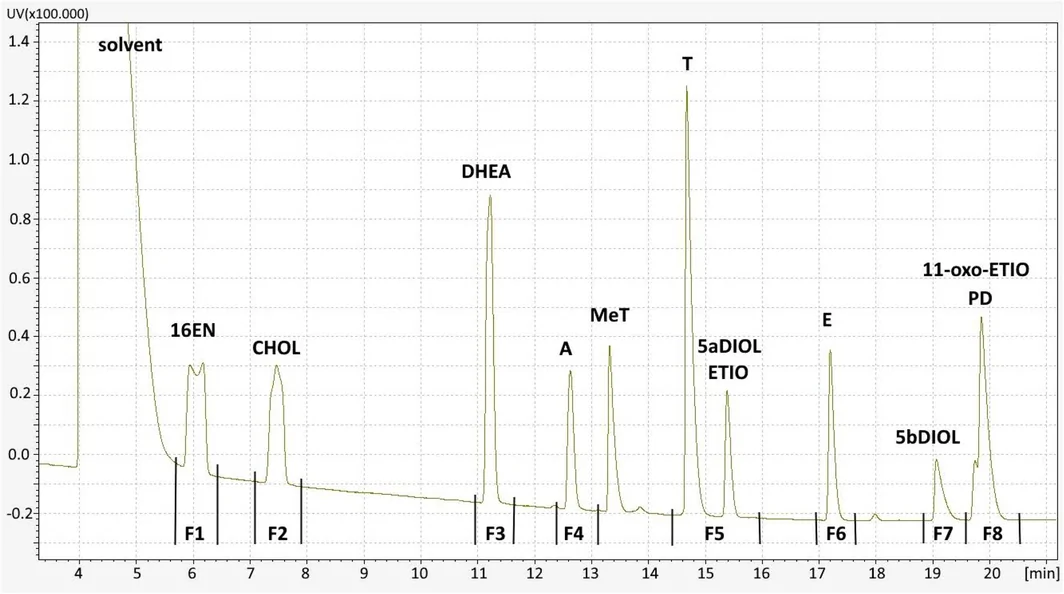
UV‐chromatogram (195 nm) of a standard mixture run before each batch of samples to fix fraction collection times. The amount of steroid on column was 5 μg for 16EN, 10 μg CHOL, 5 μg DHEA, 10 μg A, 5 μg MeT, 5 μg T, 10 μg ETIO, 5 μg E, 10 μg 5bDIOL, 20 μg PD and 10 μg 11‐oxo‐ETIO.

Fractions 5 (T, 5aDIOL and ETIO) and 8 (PD and 11‐oxo‐ETIO) underwent a second SFC‐based clean‐up and separation step utilizing a different column and gradient. Here, a Shimadzu Shim‐Pack UC Diol column (4.6 mm, 150 mm, 3 μm) was employed. The flow was at 3.5 mL/min and the gradient started at 92% CO_2_, changing to 75% CO_2_ in 12 min, followed by re‐equilibration at 92% CO_2_ for 2 min. Injections were again performed using 40 μL of ACN. Typical fraction collection times for F5 were from 4.50 to 5.40 min (ETIO), from 5.50 to 6.50 min (T), from 9.30 to 10.10 min (5aDIOL) and for F8 from 5.40 to 6.30 min (11‐oxo‐ETIO) and from 9.50 to 10.50 min (PD). Chromatograms are exemplarily shown in Figure 2. All analytes were collected in test tubes and evaporated to dryness as described above.

**FIGURE 2:**
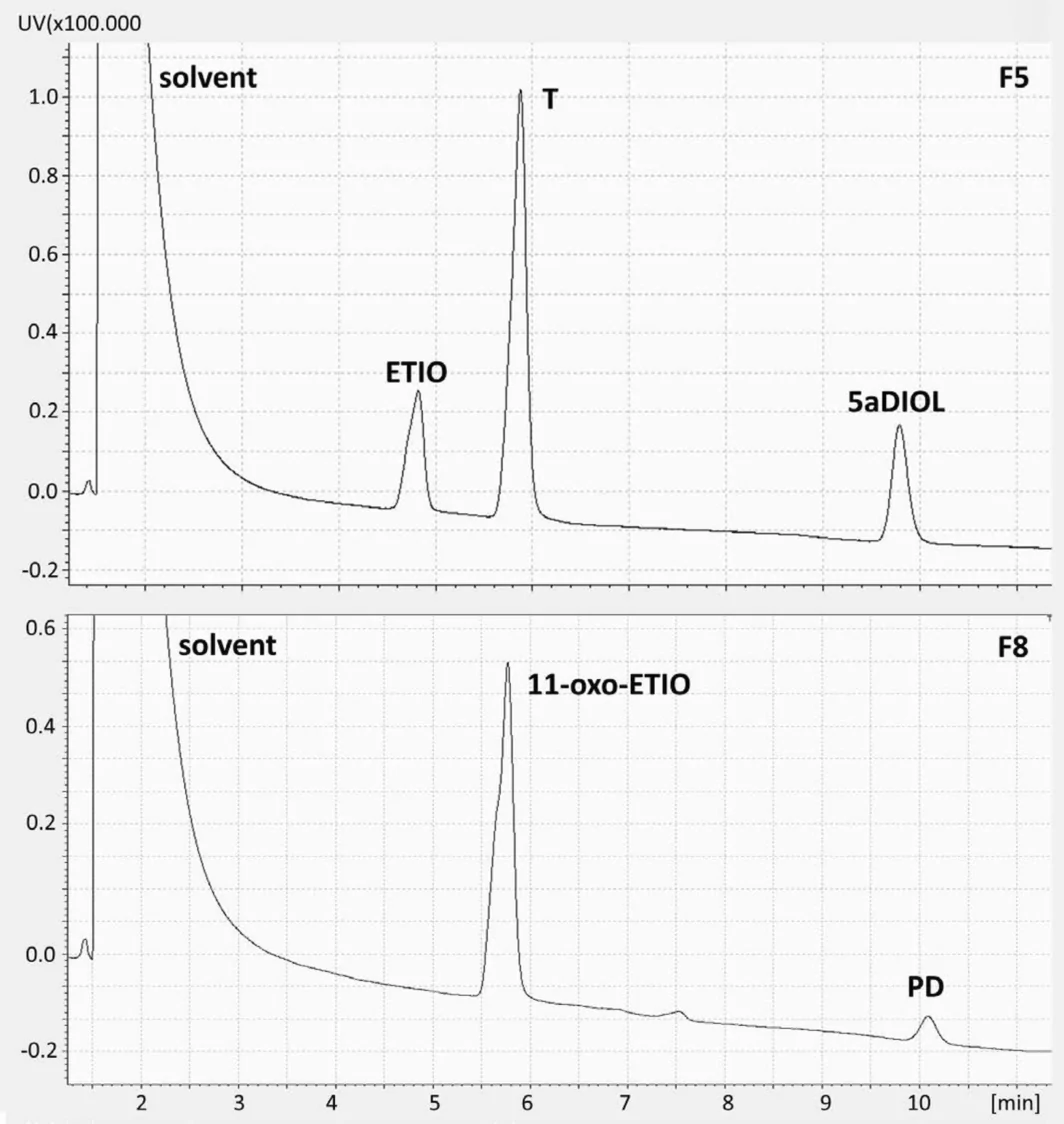
UV‐chromatogram (195 nm) of standard mixtures run before each batch of samples to fix fraction collection times for the second clean up of Fraction 5 (upper part) and Fraction 8 (lower part). The amount of steroid on column was 5 μg for T, 10 μg for ETIO and 11‐oxo‐ETIO and 20 μg for 5aDIOL and PD.

Prior to CIR determinations, all collected fractions were acetylated by adding 75 μL of pyridine and 75 μL of acetic anhydride, vortexed and incubated at 70°C for 45 min in a heating block. After derivatization, all samples were evaporated at 50°C under a gentle stream of air to dryness, reconstituted in 200 μL of MeOH and transferred to autosampler vials. Samples were dried down and reconstituted in an appropriate amount of cyclohexane—based on the respective concentration of the analytes present—ranging from 6 to 300 μL for IRMS determinations.

### 2.4 Gas Chromatography/Combustion/Isotope Ratio Mass Spectrometry

All measurements were conducted on a Delta V Plus IRMS (Thermo Fisher Scientific, Bremen, Germany) coupled to a Trace GC 1310 equipped with a TriPlusRSH Autosampler via the GC IsoLink CNH and the ConFlow IV (all Thermo). The combustion tube was operated at 950°C and kept on a constant level of oxygen by seed oxidations performed prior to each analytical run. The IRMS was hyphenated to an ISQ mass spectrometer (Thermo) via a microchannel device (SGE, Sydney, Australia) and a deactivated restriction capillary (5 m length, 0.15 mm inner diameter also from SGE) and was operated in positive electron ionization mode recording total ion chromatograms from *m/z* 50 to 500. Data were recorded and evaluated using Thermo Xcalibur software (Version 2.2).

The analytical GC column was an Agilent J&W Scientific DB‐17MS (length, 30 m; i.d., 0.25 mm; film thickness, 0.25 μm). The temperature program was based on routine conditions and only prolonged for adequate CHOL determinations [17]. It started at 100°C held for 1.5 min, then increased with 40°C/min–271°C, then with 2°C/min–305°C and then with 40°C/min to the final temperature of 320°C, which was held for 3 min resulting in a total run time of 30 min including cool‐down and equilibration. Injections were done in splitless mode at 280°C using a programmed flow starting with 2.7 mL/min held for 1.5 min and changed to 1.8 mL/min for the analytical run. The injection volume was 4 μL plus 1 μL of a solution containing 40 μg/mL RSTD in cyclohexane. Data were acquired and evaluated using Isodat 3.0 (Thermo).

### 2.5 Gas Chromatography/Mass Spectrometry

Measurements regarding the semi‐quantification of endogenous steroids were conducted on a Trace 1310 GC coupled to a Thermo TSQ 8000 Evo mass spectrometer (both Thermo). An Agilent HP‐Ultra 1 GC column (length, 17 m; i.d., 0.2 mm; film thickness, 0.11 μm) was used for chromatographic separation employing a temperature program starting at 183°C, increased with 3°C/min–232°C, then with 40°C/min to the final temperature of 310°C and held for 2 min before cooling. The injections of 1.5 μL were performed in split mode (split ratio of 1/10) at a temperature of 300°C. The system was run in multiple reaction monitoring mode, and all data were acquired and evaluated using Xcalibur (Version 4.0, Thermo).

### 2.6 Method Validation

The developed method was validated according to the requirements of the current WADA Technical Document [3].

#### 2.6.1 Linearity of the Ion Source and the Instrument

The linearity of the ion source was tested prior to each sequence by measuring 7 reference gas pulses at different peak height covering a range from 400 to 7000 mV for *m/z* 44. The linearity of the instrument was determined by repeated injections (*n* = 4) of standards at 8 different concentration levels ranging from ca. 2 μg/mL to 200 μg/mL, reflecting absolute concentrations of 8–800 ng of steroid on column. The found δ‐values were plotted against the measured peak heights and the lower limit of instrument linearity was defined by the lowest level of steroid injected showing acceptable results, i.e., the replicate determinations showed a standard deviation (SD) below 0.5‰ and the mean value at this concentration level was less than 0.5‰ different from the mean value of all determinations found in the linear range.

#### 2.6.2 Limit of Quantification (LOQ)

The LOQ of the method was determined by repeated preparations (*n* = 3) of a negative urine (QCN, *vide infra*) with known endogenous steroid concentrations at different urine volumes ranging from 5 to 0.1 mL to determine the typical LOQ for a urine aliquot of 20 mL. The LOQ was defined as the lowest urinary concentration resulting in acceptable SDs and falling into the linear range of the instrument.

#### 2.6.3 Specificity of the Method

The specificity was monitored using the ISQ mass spectrometer hyphenated to the IRMS. During method development, the mass spectra of all analytes of interest were inspected to ensure peak purity and the absence of co‐eluting compounds, which might compromise valid IRMS determinations. During the analysis related to the athlete reference population, mass spectra were investigated if the IRMS chromatogram showed any potential co‐elutions or disturbed peak shapes in the ion trace of *m/z* 44 and/or affected isotopic swings built by the ratio of extracted ion traces of *m/z* 45 over *m/z* 44.

#### 2.6.4 Negative Urine

The negative urine (QCN) used in all validation studies was a pooled urine sample of a volume of 2 L collected by one male individual over the time course of 3 days. As not all TCs and ERCs under investigation were quantified in the routine screening for the steroid profile, the standard addition technique was used to estimate the basal endogenous concentrations of all steroids. Therefore, five aliquots of 2 mL of the QCN were fortified with increasing amounts of all analytes under investigation and prepared together with a native QCN as described above. No SFC‐based purification was employed but the residue, after drying down the sample, was derivatized using 100 μL of MSTFA/NH_4_I/ethanethiol 1000/2/3 (v/w/v) and incubating the sample for 20 min at 60°C. The samples were transferred to autosampler vials and analyzed employing Gas Chromatography/Mass Spectrometry.

#### 2.6.5 Short‐ and Long‐Term Precision

The short‐term precision of the CIR of the overall method was evaluated by a six‐fold preparation and measurement of the QCN in one batch. For the long‐term precision all preparations of the QCN (*n* = 9) were considered that were produced with each batch of samples prepared for the reference population‐based study over a time period of 3 months.

#### 2.6.6 Linear Mixing Models (LMM)

In order to test for potential isotopic fractionation caused by the novel SFC‐based separation and collection of steroids under investigation, LMM were applied [6, 9].

In brief, three times six negative urine samples were prepared of which five samples were fortified with increasing amounts of all steroids under investigation, covering a range of 0.4 to 2 times the endogenous concentration, prepared batch‐wise, and analyzed. As standards with known CIR (i.e., in‐house reference materials) were used in this experiment, the obtained results directly allowed to estimate the bias introduced by the sample clean‐up. Additionally, the results were used to calculate the measurement uncertainty (MU) by employing the Gaussian least square fit to the results for each steroid [6, 9].

#### 2.6.7 Measurement Uncertainty

The measurement uncertainty was calculated based on the results obtained by the LMM using the propagation of errors. Considered were the Gaussian least square fit residuals for the intercept, the slope and the found bias based on the equation:

$$\mathrm{MU}=\sqrt{\mathrm{SD}{\left(a\right)}^{2}+\mathrm{SD}{\left(b\right)}^{2}+{\text{bias}}^{2}}$$

### 2.7 Athlete Population

The reference population encompassed 40 female and 40 male athlete routine samples collected by the National Anti‐Doping Agency of Germany (NADA) in the year 2023 and were stored frozen at −18°C until repreparation. All samples were screened for doping substances by routinely applied initial testing procedures and found to be negative, and all athletes agreed to the use of their samples for research. Additionally, in parallel to each urine sample, NADA collected DBS from each athlete, which were also screened for doping agents and found to be negative. The DBS were also stored at −18°C until analysis. Although the urine samples were used to evaluate the fitness‐for‐purpose of the new SFC‐based method considering a large variety of different biological matrices and endogenous urinary concentrations for all TCs and ERCs, the DBS were solely analyzed for the CIR of CHOL.

### 2.8 DBS

In order to extract CHOL from the DBS, the routinely applied sample preparation protocol was slightly adapted [21, 22]. In brief, the Tasso polymer or an 8 mm punch of the cellulose‐based sample card were extracted with 400 μL of MeOH in an ultrasonic bath for 20 min. After extraction, the samples were centrifuged at 13.3*g* for 5 min and the supernatant was transferred to a test tube and evaporated to dryness at 50°C using a gentle stream of air. Then, acetylation was conducted as described above; the dried residues were transferred to autosampler vials using 300 μL of cyclohexane, and the injection volume was 4 μL plus 1 μL of a solution containing 40 μg/mL RSTD in cyclohexane.

### 2.9 Administration Trial

One male volunteer (51 years, 180 cm, 82 kg) administered 100 mg of testosterone gel (two 50 mg pouches of TESTOGEL, Dr. Kade/Besins Pharma GmbH, Berlin, Germany) for five consecutive days in the morning. Negative urine samples (*n* = 7) were collected on the 3 days prior to the first application. During the application, four samples per day were collected, the first in the morning prior to the next administration and then one sample approx. every 4–5 h. After the last administration all urine samples were collected for 35 h. The samples were stored frozen at −18°C until analysis.

The volunteer gave written consent, and the study was approved by the local ethics committee of the German Sport University Cologne (ethical_approval_130404).

### 2.10 Chromatographic Isotopic Fractionation and SFC

A strong chromatographic isotopic fractionation has been described for steroidal compounds separated on reverse‐phase columns using HPLC [9, 23]. In order to elucidate whether this phenomenon is also present using SFC and a normal‐phase HILIC column, different steroid standards (CHOL, T, PD) were injected at elevated concentrations of up to 50 μg steroid on column and narrow (12–15 s wide) fractions were collected over each individual analyte peak. Additional peak broadening was achieved by removing the pre‐column solvent mixer. All collected fractions were evaporated, acetylated, and forwarded to IRMS to monitor the shift in CIR over the entire chromatographic peak.

## 3 Results and Discussion

Publications on the use of SFC to quantify or clean up endogenous urinary steroids and steroid‐conjugates are scarce [24, 25, 26]. Regarding the quantification, both human and bovine urine samples were investigated and methods have been developed [24, 26]. The clean‐up of urinary steroids has been investigated but only preliminary data were obtained and the approaches were not fully validated [25, 26]. Therefore, the full validation in line with current regulations was one of the main tasks of this work in order to present a fit‐for‐purpose method.

### 3.1 Efficiency of the SFC‐Based Clean‐Up

In order to ensure valid results, all analytes have to be sufficiently clean for IRMS determinations. In Figure 3, chromatograms obtained on the QCN are exemplarily shown to demonstrate the clean‐up performance of SFC. For the majority of analytes, only the respective ERC or TC is present in significant amounts in the corresponding collected fraction. This enables valid determinations also for urinary steroids showing significantly different concentration since dilution can be adopted as needed. Regarding 5aDIOL, in most of the investigated samples another endogenous steroid was co‐collected with 5aDIOL, androst‐5‐ene‐3β,17β‐diol (5ENDiol). This steroid is a metabolite of DHEA and, in parallel to DHEA, predominantly excreted sulfoconjugated with only minor amounts found in the free and glucuronoconjugated fractions [27, 28]. Therefore, the found concentrations were always comparable to 5aDIOL and did not hamper valid CIR determinations.

**FIGURE 3:**
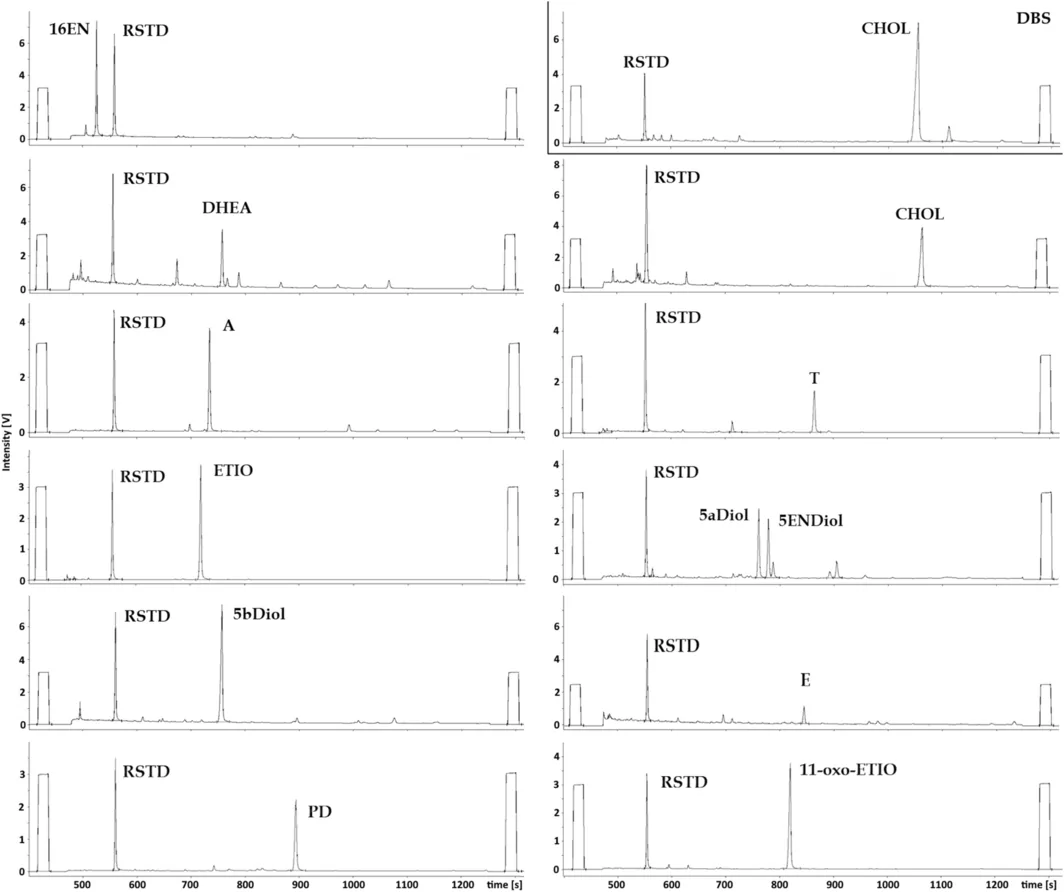
IRMS chromatograms (*m/z* 44) of all ERCs and TCs extracted from the QCN demonstrating the efficient sample clean‐up of the novel method. On the upper right, the chromatogram obtained for CHOL extracted from a typical DBS sample is shown. All steroids were analyzed acetylated.

### 3.2 Method Validation

Considering that the developed method should be applicable in sports drug testing, the complete method validation was based on the current regulations for IRMS in doping controls [3].

#### 3.2.1 Linearity of the Ion Source and the Instrument

The linearity of the ion source was always within the manufacturer's specification. The found linear range of the employed IRMS system was comparable to earlier reported results and listed here in Table 1 [17, 29]. This parameter does not depend on the sample preparation and therefore this parallelism could be expected.

**TABLE 1:** Results obtained on the linear range of the IRMS giving the minimum and the maximum peak height and corresponding estimated limits of quantification for a 20 mL aliquot.

Steroid	Linear range [mV peak height]		LOQ [ng/mL]
16EN	135	6400	13
CHOL	110	5700	31
11‐oxo‐ETIO	470	6500	32
PD	117	9300	17
DHEA	125	5700	3
ETIO	150	7300	47
A	194	12,400	56
E	155	7500	6
5bDIOL	170	11,700	4
5aDIOL	130	9000	6
T	280	7400	3

#### 3.2.2 LOQ

The found LOQ essentially represents a combination of the found linear range and the recovery of the method and therefore depends on the applied sample clean‐up protocol. Considering that the wet chemistry protocol encompassing solid‐phase and liquid–liquid extractions was not adapted, possible changes in the LOQ should be driven solely by the SFC‐based fractionation. As shown in Table 1, again comparable results were obtained to other in‐house validated methods and all requirements as per TD2022IRMS were fulfilled [3, 29, 30].

#### 3.2.3 Specificity of the Method

During method development relying mainly on the QCN as biological matrix, no co‐eluting peaks were visible. The fractions containing DHEA and E showed increased background noise (see Figure 3) compared to other chromatograms, mainly due to the low endogenous urinary concentrations of these analytes and the resulting low volumes of reconstitution solvent (6–2 μL of cyclohexane) prior to injection. Regardless, the target analytes were clean and not affected by any co‐elutions. Within the reference population encompassing 40 female and 40 male samples, only the determinations of E were partly hampered (*n* = 17) by low urinary concentrations and co‐elutions whereas the measurements of DHEA were not affected at all. Considering that low urinary concentrations of E are usually not investigated in the context of sports drug testing (only samples showing elevated concentrations of > 50 ng/mL in females and > 200 ng/mL in males are forwarded to IRMS), the results were considered to be fit‐for‐purpose [3].

#### 3.2.4 Short‐ and Long‐Term Precision

The results obtained on the inter‐ and intra‐day precision are summarized in Table 2. In line with earlier investigations, the found SDs within one batch were significantly lower for most of the steroids compared to the in‐between variations [31]. Especially the lower concentrated steroids (DHEA and E) were affected by slightly larger SDs.

**TABLE 2:** Inter‐ and intra‐day precision found for the QCN.

	16EN	CHOL	11‐oxo‐ETIO	PD	DHEA	ETIO	A	E	5bDiol	5aDiol	T
6‐Fold preparation of the QCN in one batch											
Mean	−22.6	−23.0	−22.6	−23.6	−23.0	−23.1	−22.5	−25.7	−23.9	−23.0	−24.9
SD	0.19	0.18	0.25	0.36	0.12	0.08	0.15	0.22	0.19	0.44	0.31
9‐Fold preparation of the QCN with each batch of the reference population											
Mean	−22.6	−22.8	−22.4	−23.1	−22.8	−23.0	−22.2	−25.0	−23.5	−23.3	−24.0
SD	0.42	0.59	0.64	0.63	0.67	0.40	0.17	0.71	0.54	0.49	0.46

#### 3.2.5 LMM and Calculated Measurement Uncertainties

The results obtained on the LMM for all steroids under investigation are summarized in Table 3. All results were comparable to earlier published results [6, 15, 17]. The criteria defined as per TD2022IRMS are met for all analytes [3].

**TABLE 3:** Results obtained on the linear mixing model for all steroids under investigation.

	16EN	CHOL	11‐oxo‐ETIO	PD	DHEA	ETIO	A	E	5bDIOL	5aDIOL	T
a	5.9	2.0	−6.2	4.7	6.1	−1.4	6.5	7.3	3.8	−2.9	4.5
SD (a)	0.29	0.27	0.44	0.49	0.40	0.24	0.28	0.35	0.15	0.48	0.38
b	−32.9	−28.0	−20.6	−34.7	−32.8	−26.2	−33.4	−36.1	−34.5	−27.2	−32.5
SD (b)	0.17	0.18	0.27	0.28	0.28	0.14	0.18	0.23	0.10	0.29	0.22
Std	−32.5	−27.7	−20.6	−34.2	−32.9	−26.3	−33.6	−36.2	−34.7	−27.2	−32.8
Bias	0.43	0.13	0.33	0.40	0.37	0.13	0.19	0.39	0.55	0.37	0.32
MU	0.55	0.35	0.61	0.69	0.61	0.31	0.39	0.57	0.58	0.67	0.54

### 3.3 Athlete Population

Besides establishing SFC as a sample clean‐up technique in IRMS of doping control samples, a new ERC (CHOL) was also under investigation here. In order to provide a sound basis for evaluation of this novel ERC, it was decided to expand the investigated reference population. According to the TD2022IRMS, only 20 male and 20 female samples are considered to be sufficient to demonstrate the suitability of the method [3]. In line with earlier investigations, a number of 60–80 samples were thought to be sufficient for a first estimation resulting in the *n* = 80 samples included in this study [6, 7, 8, 16, 17, 20, 28, 31, 32, 33, 34, 35]. In addition to the results obtained on urinary steroids, CHOL was also investigated in DBS, which were collected in parallel to the employed urine samples.

#### 3.3.1 Reference Population—Urine

In Figure 4, the results obtained for all steroids and their respective δ^13^C_VPDB_‐values are shown. Again, the found CIR were directly comparable to earlier findings of reference populations investigated in Central Europe [7, 31].

**FIGURE 4:**
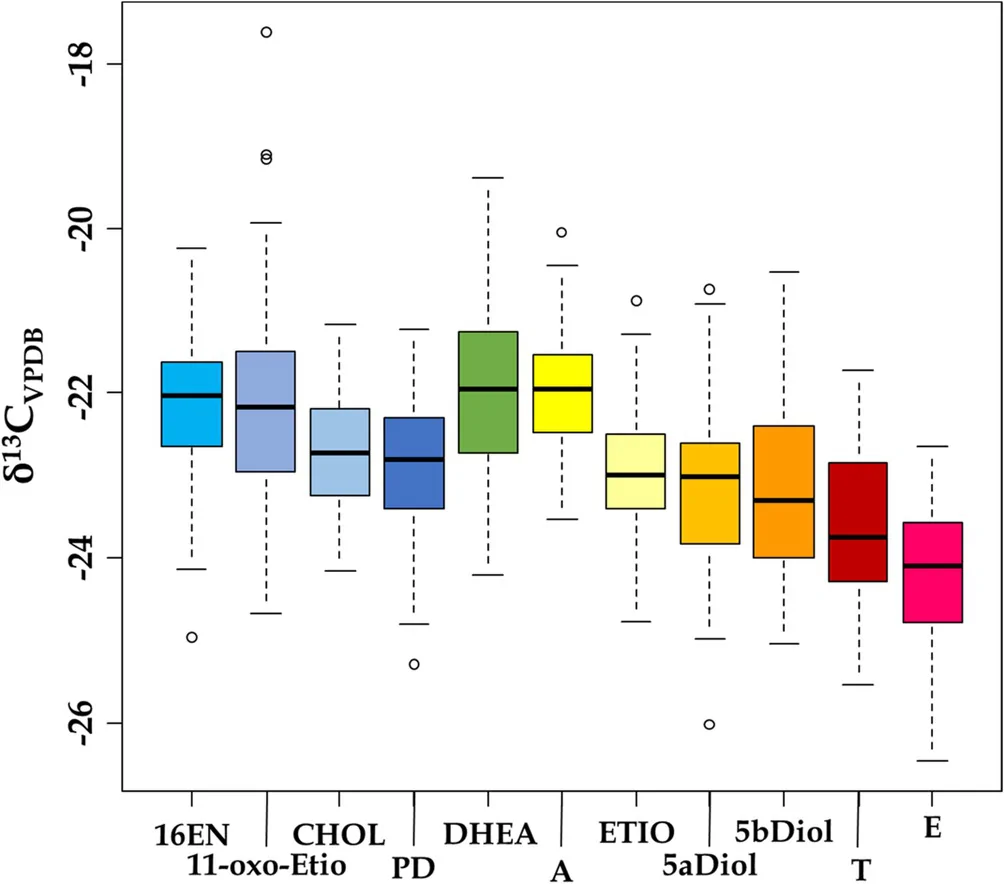
Boxplots representing the absolute δ^13^C_VPDB_‐values found in the investigated reference population encompassing 40 female and 40 male athletes. Abbreviations: 11‐oxo‐Etio, 11‐oxo‐etiocholanolone; 16EN, 16‐androstenol; 5aDiol, 5α‐androstanediol; 5bDiol, 5β‐androstanediol; A, androsterone; CHOL; cholesterol; DHEA, dehydroepiandrosterone; E, epitestosterone; ETIO, etiocholanolone; PD, pregnanediol; T, testosterone.

Small differences in the mean values of different TCs or ERCs can be expected and are mainly due to isotopic fractionation during the metabolism of steroids. For doping controls, the Δ‐values are more important and these are listed in Table 4. For all pairs of ERC‐TC, the criteria as per TD2022IRMS were fulfilled with the exception of 11‐oxo‐ETIO minus T [3]. In general, the Δ‐values built with 11‐oxo‐ETIO showed slightly elevated SDs compared to other ERCs and this was mainly due to three potential outliers also visible in Figure 4. The CIR for these measurements of 11‐oxo‐ETIO fall between −19‰ and −17‰ and are therefore more enriched than other ERCs. One possible explanation would be the administration of cortisone, which may show enriched CIR in some preparations and of which 11‐oxo‐ETIO would represent a urinary metabolite [33, 36, 37]. Deleting the enriched values from the reference population improves the SDs, but as no clear criteria exist to define a potential administration of cortisone, it was decided to keep the enriched values in the population.

**TABLE 4:** Δ‐values for all combinations of ERCs and TCs found in the reference population encompassing 40 female and 40 male athletes.

ERC	TC							
	DHEA	ETIO	A	E	5bDIOL	5aDIOL	T	
PD	−0.74	0.18	−0.83	1.46	0.36	0.35	0.86	Mean
	0.91	0.67	0.63	0.79	0.94	0.80	0.93	SD
CHOL	−0.69	0.25	−0.77	1.54	0.41	0.41	0.90	Mean
	0.74	0.77	0.69	0.86	0.88	0.91	0.66	SD
11‐oxo‐ETIO	−0.23	0.69	−0.32	1.97	0.89	0.87	1.40	Mean
	0.96	0.67	0.75	0.87	0.98	0.96	1.11	SD
16EN	−0.15	0.80	−0.19	2.14	0.94	0.95	1.46	Mean
	0.79	0.62	0.53	0.70	0.97	0.79	0.77	SD

From the overall *n* = 880 measurements regarding the population‐based studies, only 30 measurements were not accomplished (3.4%). As mentioned before, E was not measurable in *n* = 17 samples mainly due to co‐elutions and low urinary concentrations, T concentrations were too low in nine female samples and 16EN concentrations in four female samples. The results demonstrate the fitness‐for‐purpose of the developed method in the context of doping controls.

#### 3.3.2 Reference Population—DBS

The determination of CHOL was successful in all 80 urine and DBS samples investigated in course of the study. As CHOL is found in blood in significant amounts, the small sample size of DBS did not hamper the valid measurements, and as no other steroidal compound is present at comparable concentrations, no further sample clean‐up was necessary here. An exemplary chromatogram is shown in Figure 3 in the upper right part. The small peak eluting after CHOL at 1115 s was tentatively identified as cholesta‐3,5‐dien‐7‐one and seems to represent a degradation product of CHOL only visible in stored DBS. Neither freshly collected and prepared DBS nor serum samples showed this peak.

The CIR of CHOL found in urine and DBS was significantly different with a mean difference of 0.58‰ (Figure 5A). This offset resulting in more enriched values in DBS was relatively stable and resulted in a narrow distribution for the difference calculated for CHOL (DBS)‐CHOL (urine) as shown in Figure 5B. With 0.53‰, the SD for all pairs of CHOL in the reference population was only slightly higher than the theoretical SD considering a MU of 0.35 as found for urinary CHOL (Table 3). Small differences between circulating and urinary excreted steroids can be expected and have been substantiated for A, for example (unpublished results). Likewise, for urinary steroids, depending on their phase‐II metabolism, differences were found for the same steroid excreted glucuronoconjugated and sulfoconjugated [15, 31]. These small differences can be attributed to metabolic isotopic fractionations and do not exclude the use these steroids in sports drug testing. Therefore, the results obtained for urinary CHOL enable the use of this new ERC in parallel to other well‐established ERCs. This is also substantiated by the found Δ‐values (Table 4), which fall well between those obtained for PD and 11‐oxo‐ETIO, the primary and secondary ERC used in the routine doping control analysis in the Cologne laboratory.

**FIGURE 5:**
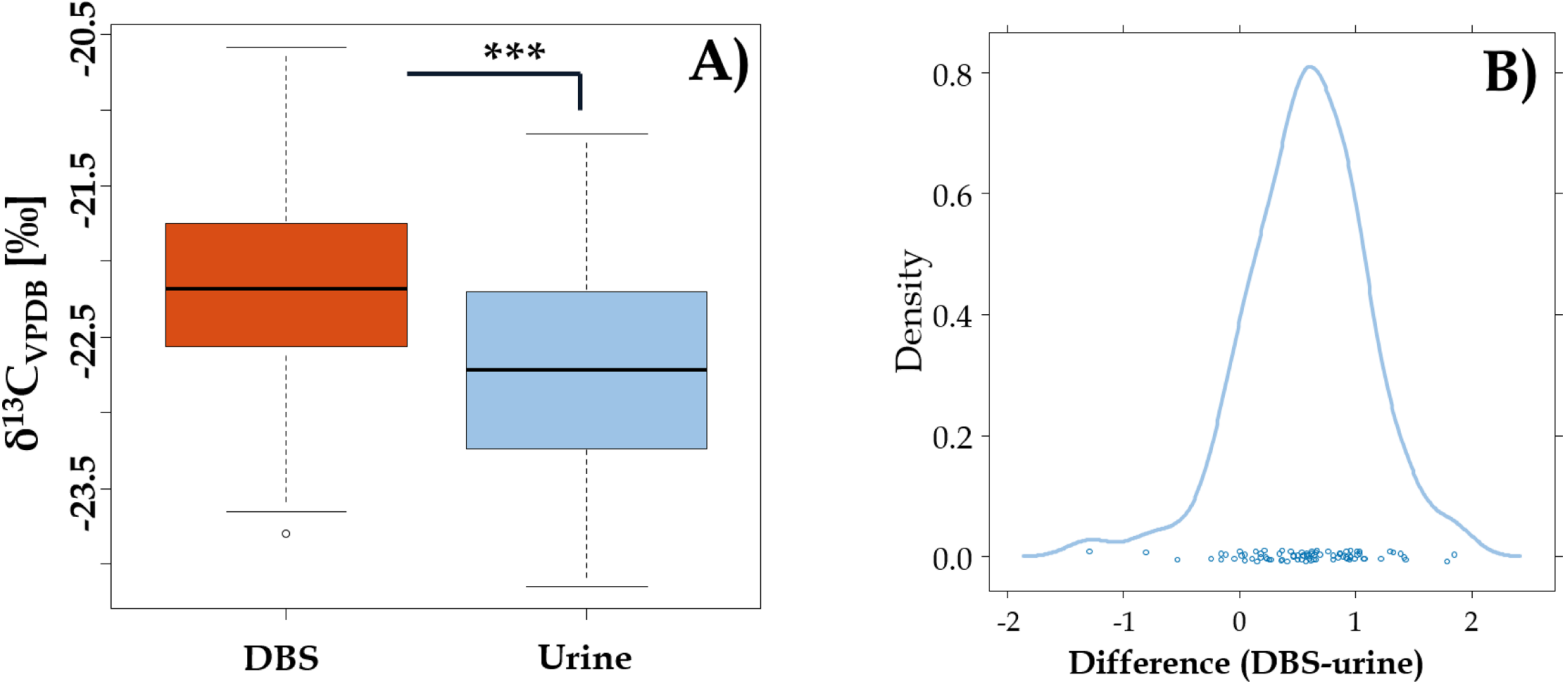
Comparison of CIR values of CHOL obtained in DBS and urine. (A) Boxplot of absolute values found in the reference population encompassing *n* = 40 females and *n* = 40 males, ***p < 0.001. (B) Density plot of the found differences between CHOL derived from DBS and urine.

### 3.4 Administration Trial

The influence of testosterone gel on the CIR of endogenous urinary steroids has already been described in literature several times and was here mainly re‐investigated as proof‐of concept of the new SFC‐based sample preparation [6, 35, 38, 39, 40, 41]. The main findings are perfectly in line with expected values and are depicted in Figure 6. The urinary T was directly influenced after the topical administration and the determined CIR was close to that of the administered T, which was found to have an isotopic composition of δ^13^C_VPDB_ = −29.8‰ ± 0.23‰ (*n* = 6). Due to the 5α‐reductase activity in the human skin, the T‐metabolite 5aDIOL was significantly affected by T‐gel administrations and was found more depleted than T itself. The other T‐metabolite encompassing a 5α‐configuration (A) showed less depleted CIR, and the impact of the exogenous T on its CIR was much slower, mainly due to the larger pool size of circulating A. As A is additionally a metabolite of DHEA and constantly endogenously produced even if T is administered, the CIR of A hardly reached that of the exogenous T.

**FIGURE 6:**
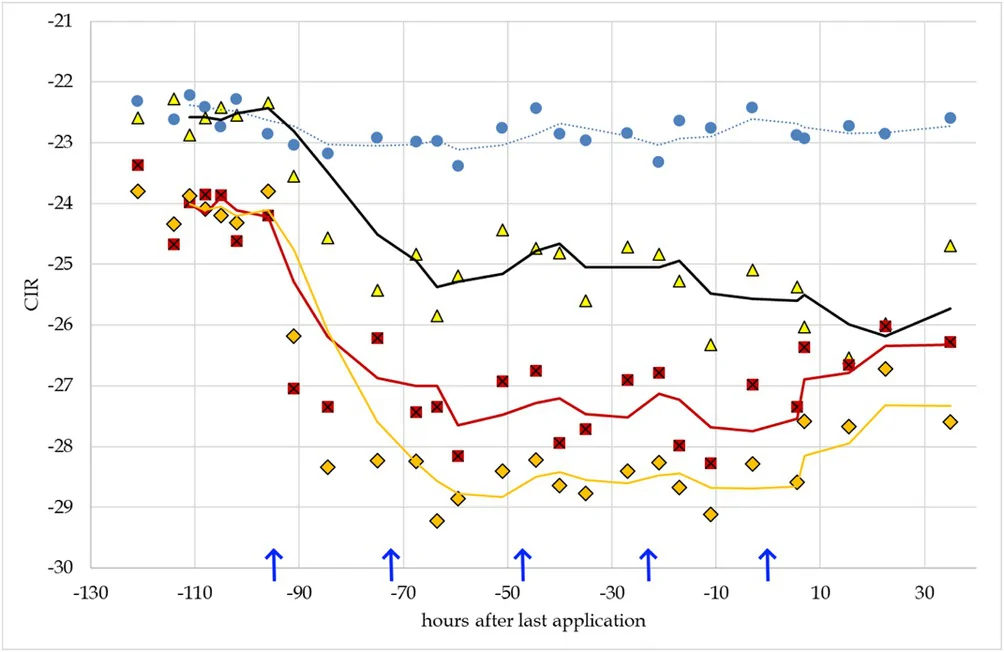
Carbon isotope ratios (δ^13^C_VPDB_) of selected urinary steroids after the five‐fold administration of 100 mg of testosterone gel. Each timepoint of administration is highlighted by a blue arrow. Blue circles stand for PD, yellow triangles for A, red squares for T and orange diamonds for 5aDIOL. The respective lines represent the moving average.

An interesting effect of the prolonged administration of T on urinary E is shown in Figure 7. Usually, it is assumed that the interconversion of T to E is negligible but in principle existing in humans [42]. Especially 4‐androstene‐3,17‐dione (A4) acts as a possible precursor for both T and E [43]. Although the interconversion of A4 and T is constantly taking place in the human body, the conversion of A4 to E is considered to be significantly smaller compared to T as the equilibrium of this reaction is expected to be mostly on the side of A4 (Figure 8).

**FIGURE 7:**
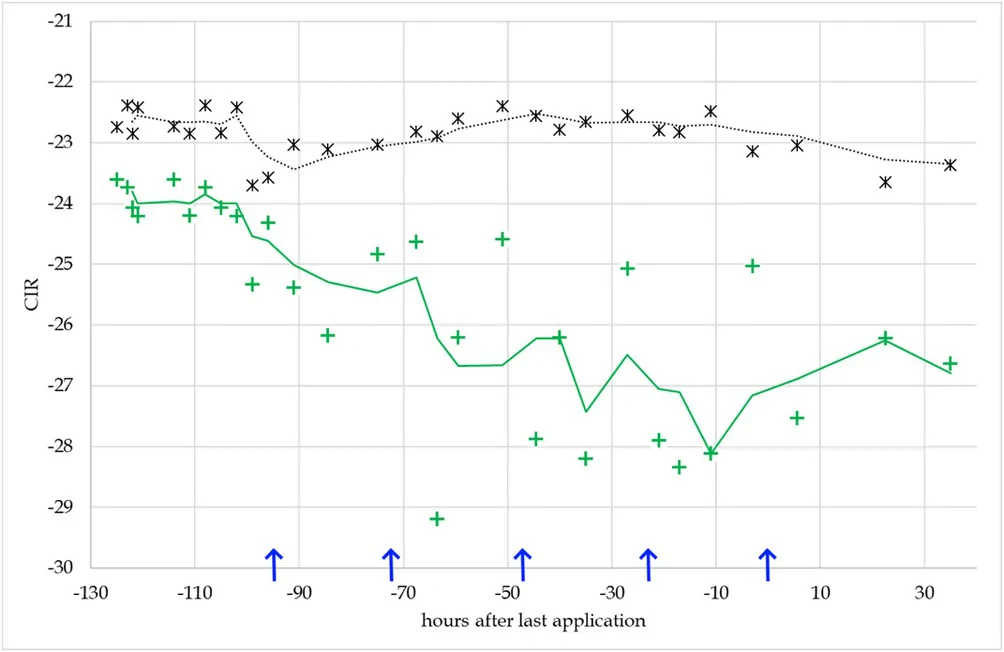
Carbon isotope ratios (δ^13^C_VPDB_) for CHOL (black crosses) and E (green plusses) determined after the five‐fold administration of 100 mg of testosterone gel. Each timepoint of administration is highlighted by a blue arrow.

**FIGURE 8:**
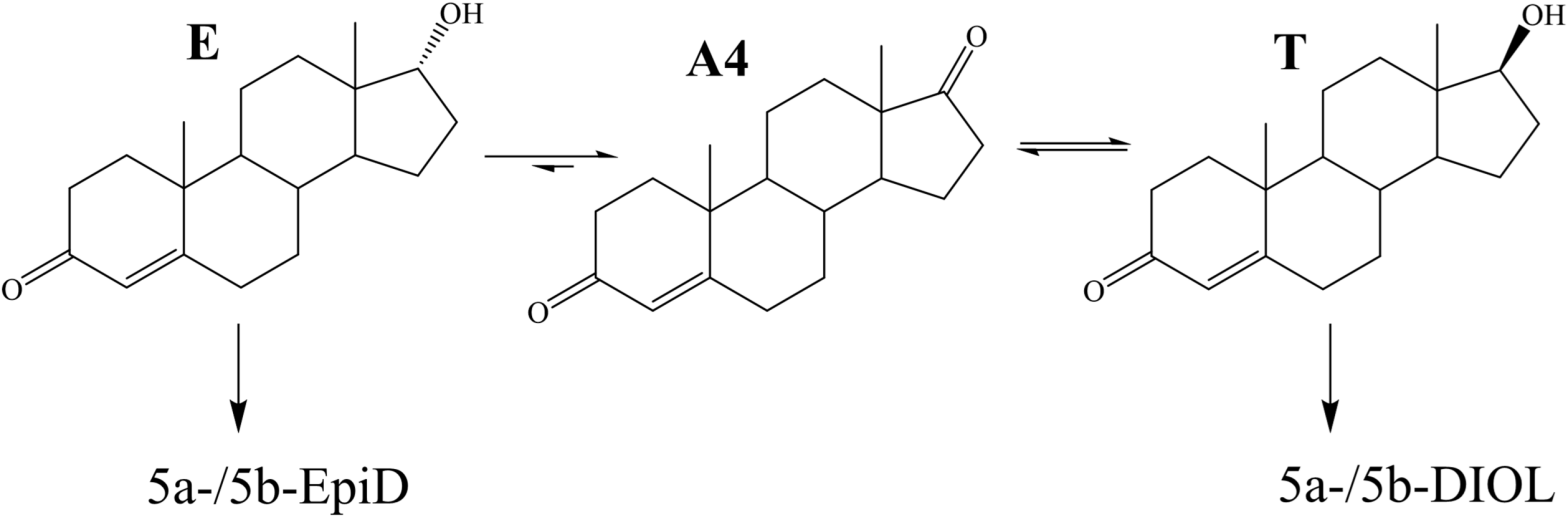
Metabolic fate of 4‐androstenedione (A4) driven by 17α‐ and 17β‐hydroxysteroid dehydrogenase and subsequent conversion of the intermediates (E and T) to their corresponding androstanediols. 5a‐ and 5b‐EpiD stand for 5α‐ and 5β‐androstane‐3α,17α‐diol.

Although single administrations of T do not show any significant influence on the production and excretion of E, it is well known that multiple administrations of T decrease the excretion of E as the endogenous production of all androgens is downregulated [44]. As soon as the endogenous production of E is decreasing, the relative amount of E derived from A4 is increasing, resulting in a traceable conversion of T to E. As soon as this conversion becomes predominant, the CIR of E will reflect those of T. This potential phenomenon was already detected in routine doping control samples from time to time. For example, in a bodybuilder's urine sample collected after admitted constant T administrations for 4 weeks, both T and E showed similar CIR with δ^13^C_VPDB_ = −30.1‰ and −30.5‰, respectively (unpublished results). This demonstrates that the small amount of E still excreted into urine was completely derived from the exogenous T by interconversion, most probably via A4.

The results obtained in this study after the five‐fold administration of T‐gel perfectly substantiate the hypothesis of the described interconversion (Figure 7). In the course of the study, the CIR of E showed more and more depleted CIR getting closer and closer to the depleted values of the administered T. The found CIR were more variable compared to other investigated TCs and especially in the urine samples collected in the morning before the next administration of T‐gel the values were found close to the endogenous starting values again. Nevertheless, these results clearly indicate that depleted values of E cannot directly be attributed to the administration of E but may be due to endogenous interconversion of T or T‐prohormones especially after prolonged administrations.

### 3.5 Chromatographic Isotopic Fractionation and SFC

The influence of chromatographic isotopic fractionation during the HPLC‐based sample clean‐up can be significant and invalidate the complete analysis as either only TCs or ERCs may be affected resulting in false positive or false negative results depending on which steroid was influenced [9, 23]. Therefore, the impact of SFC‐based chromatography on selected steroids was investigated and the results are presented in Figure 9.

**FIGURE 9:**
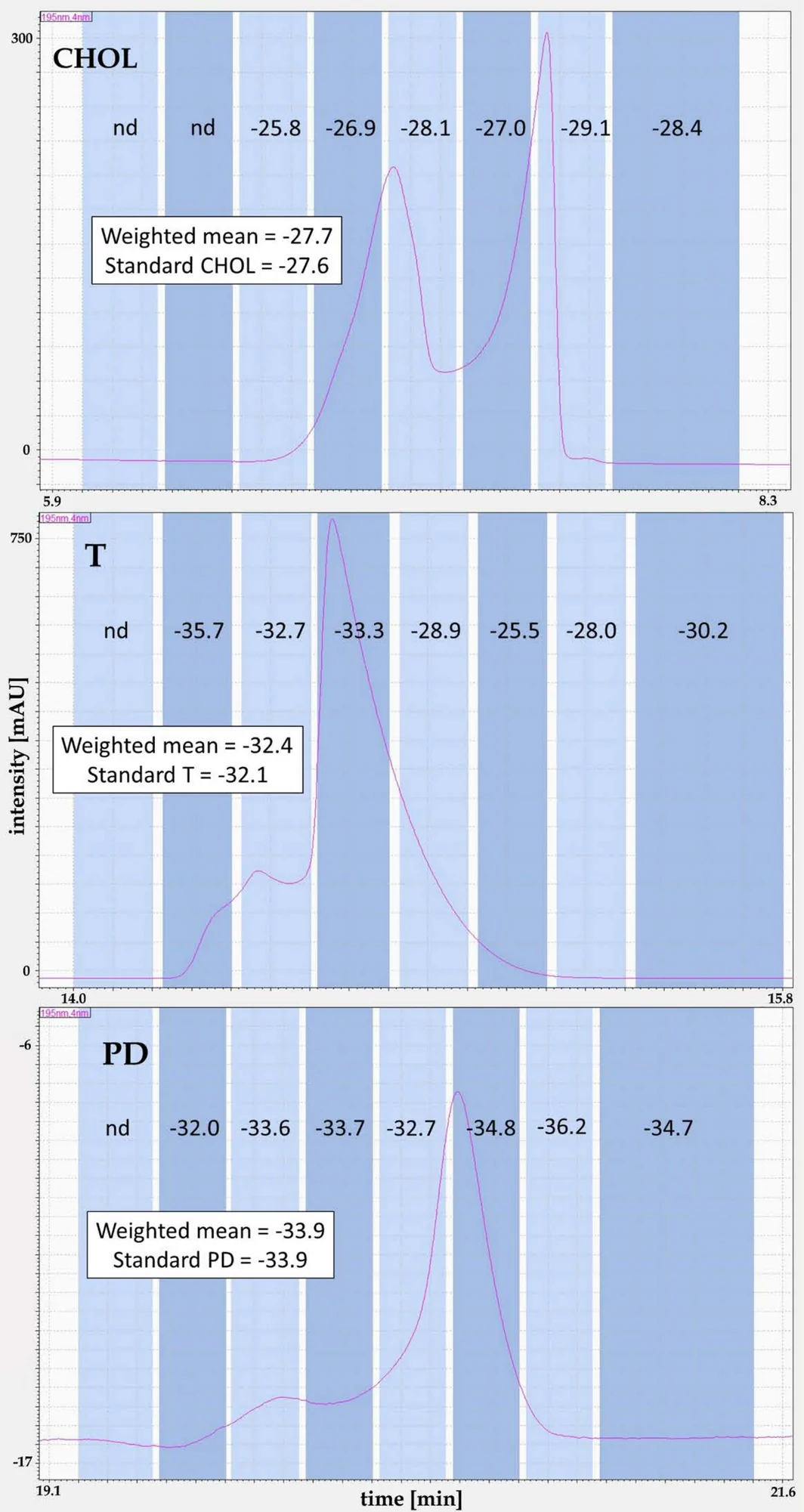
Chromatographic isotopic fractionation observed on the SFC system. Light and dark blue boxes represent the collected fractions, the numbers are the determined δ^13^C_VPDB_‐values of each fraction. Further information in the text.

In order to cover the complete range of retention times, the early eluting CHOL, the late eluting PD, and T eluting in‐between were investigated exemplarily. As expected, different parts of each peak showed slightly different CIR. The distribution of δ^13^C_VPDB_‐values was not as uniformly as found on HPLC systems, where the peak tends to be more enriched at the beginning and more depleted at the end [9, 23]. The more random scatter of the isotopic composition found here may be attributed either to the distorted peak shape of each analyte or to the use of a different LC column compared to the conventional C‐18 material used for reverse phase HPLC. The source of this phenomenon could not be unambiguously identified and may be addressed in future research. Regarding the use of SFC in routine doping controls, the main finding was that if the weighted mean of all collected fractions and the belonging isotope ratios was calculated, the mean value was perfectly in line with the CIR of the used standard. As described for HPLC, collecting the complete chromatographic peak is a prerequisite for valid CIR determinations and has to be ensured during sample preparation. As done in current routine applications it seems to be favorable to add a safety margin of approx. 0.3 min to the starting point and to the end of fraction collection ensure this prerequisite is met.

## 4 Conclusions

The applicability of SFC‐based sample clean‐up using fraction collection in the context of CIR‐determinations in sports drug testing was investigated for the first time. The developed method was fully validated considering current WADA regulations and fulfilled all requirements as per TD2022IRMS. Obtained results were highly comparable to well‐established approaches using HPLC‐based fraction collection. In contrast to conventional sample preparation, SFC allowed for a faster chromatographic separation of TCs and ERCs, resulting in shorter run times and especially the necessary evaporation of collected fractions prior to GC/C/IRMS measurements was accelerated and found to be approximately five times faster.

As a novel ERC (CHOL) was investigated for the first time, a reference population of *n* = 80 female and male athletes was employed to demonstrate the fitness‐for‐purpose of the new method and to enable in‐depth investigations on the new ERC. As the suitability of urinary CHOL as ERC was previously under discussion, DBS collected from the athletes included in the reference population during the same sample collection event as for urine were also analyzed for CHOL. Albeit showing a systematic offset between urinary and circulating CHOL, the difference was highly reproducible and therefore pointed towards a general metabolic isotopic fraction, which will not hamper the use CHOL as ERC in doping control analysis. Especially as all CIR found for urinary CHOL were well within the range of the other investigated ERCs.

The investigated administration trial encompassing a five‐fold application of T‐gel demonstrated again the satisfactory comparability between the classical HPLC and the new SFC‐based approach. An interesting finding here was the significant influence on the CIR of urinary E demonstrating the potential interconversion of T to E. This finding will have to be considered in the result management process of atypical or adverse analytical findings dealing with depleted CIR of E as these depleted values cannot be taken as proof for the illicit administration of E (as a masking agent) if T has been administered.

Chromatographic isotopic fractionation takes place under SFC conditions as it does for HPLC clean‐up necessitating the complete collection of chromatographic peaks in order to ensure valid IRMS determinations.

SFC‐based sample clean‐up can be used in doping control analysis and speeds up the sample preparation providing comparable results as demonstrated for the classical HPLC‐based procedures.

## Funding

This work was supported by World Anti‐Doping Agency (10.13039/501100001962; 241A02MT), Manfred‐Donike Institute for Doping Analysis, and the Federal Chancellery of the Federal Republic of Germany.

## Data Availability

The data that support the findings of this study are available from the corresponding author upon reasonable request.
